# Comparative evaluation of the prophylactic activity of a slow-release insecticide collar and a moxidectin spot-on formulation against *Thelazia callipaeda* infection in naturally exposed dogs in France

**DOI:** 10.1186/s13071-015-0696-6

**Published:** 2015-02-10

**Authors:** Charlotte Lechat, Noémie Siméon, Olivier Pennant, Loïc Desquilbet, Sabine Chahory, Christophe Le Sueur, Jacques Guillot

**Affiliations:** Parasitology, Ecole nationale vétérinaire d’Alfort, Maisons-Alfort, France; Veterinary Clinic, Notre Dame de Sanilhac, France; Veterinary Clinic, Vergt, France; Biostatistics and clinical epidemiology, Ecole nationale vétérinaire d’Alfort, Maisons-Alfort, France; Ophthalmology, Ecole nationale vétérinaire d’Alfort, Maisons-Alfort, France; Bayer HealthCare Animal Health, Puteaux, France

**Keywords:** *Thelazia callipaeda*, Eyeworm, Prevention, Dog, France

## Abstract

**Background:**

The relative efficacy of a collar containing 10% imidacloprid and 4.5% flumethrin (Seresto®, Bayer HealthCare Animal Health) and a spot on formulation containing 10% imidacloprid and 2.5% moxidectin (Advocate®, Bayer HealthCare Animal Health) was evaluated as a control measure to prevent canine thelaziosis in dogs in an endemic area of France.

**Findings:**

Ninety-six privately-owned dogs were enrolled in the multicentre, controlled study. Before summer (the period of transmission by fruit flies), dogs were allocated to one of three groups: Group A (n = 36)- treated once with a collar containing 10% imidacloprid and 4.5% flumethrin; Group B (n = 33)- treated every month for 8 months with a spot-on containing imidacloprid 10% and moxidectin 2.5%; and Group C (n = 27)- untreated control animals. Dogs were regularly subjected to ocular examination in order to assess *Thelazia callipaeda* infection. During the trial, *T. callipaeda* nematodes were detected in 12 (33%) collared dogs (group A) whereas no eyeworm could be found in dogs who received a monthly spot on application of moxidectin (group B). In the control group, 8 (30%) dogs became infected.

**Conclusions:**

The monthly application of a spot on formulation containing 10% imidacloprid and 2.5% moxidectin was shown to be highly effective in preventing *T. callipaeda* infection in a population of dogs living in an endemic area in France. On the contrary, the slow-release collar tested in this study did not display any protection against canine thelaziosis.

## Findings

### Background

*Thelazia callipaeda* (Spirurida, Thelaziidae) is a nematode that lives in the conjunctival sac of domestic and wild carnivores, rabbits and humans causing mild to severe clinical signs (epiphora, conjunctivitis, keratitis and even corneal ulcers) [1]. In Europe, *Thelazia callipaeda* is transmitted by *Phortica variegata* (Diptera, Drosophilidae), a small secretophagous fly. This insect feeds on fruits, vegetables and also on lacrymal secretions of domestic animals and wildlife [2]. In the past two decades *T. callipaeda* infection was proved to be widespread among dogs and cats from northern (Aosta valley) to southern (Basilicata region) Italy [3,4]. Following the first descriptions in Italy, *T. callipaeda* has been increasingly reported in western France (Dordogne area) [5,6], Switzerland [7], Spain [8], and Portugal [9]. Recently, the first autochthonous cases of thelaziosis have been described in red foxes, dogs and a cat living in Bosnia and Herzegovina and Croatia [10]. The geographical expansion of *T. callipaeda* in previously non-endemic countries of Europe may be attributed to the dispersal of the infection with wild carnivores (especially red foxes), which are suitable hosts for this parasite and may easily move in neighbouring regions [11]. The growing number of cases of canine thelaziosis in Europe is also a consequence of the increased mobility of dogs and the absence of reliable preventive measures in pet carnivores. In a previous investigation, monthly administration of milbemycine oxime was shown to significantly reduce *T. callipaeda* infection rate in dogs in the field [12]. Milbemycine oxime is the only molecule, which has been registered in Europe for the treatment of canine thelaziosis. The molecule is used orally at 0.5 mg/kg and two administrations at one week interval are recommended. Recently, a polymer matrix collar containing a combination of 10% imidacloprid and 4.5% flumethrin (Seresto®, Bayer HealthCare Animal Health) has been licensed for use in dogs and cats. This collar conferred long-term protection against fleas and ticks [13]. It was successful in preventing the transmission of tick or flea-borne pathogens [14,15] and a study recently demonstrated its efficacy in the prevention of infection by *Leishmania infantum* in dogs in a hyper-endemic area in Italy [16].

The aim of this study was to determine whether application of the slow-release insecticide collar containing 10% imidacloprid and 4.5% flumethrin could reduce *T. callipaeda* transmission in dogs living in Dordogne, France. The prophylactic activity of a spot-on formulation containing moxidectin was also evaluated over the 9 month-study period (from May 2012 to January 2013).

### Methods

#### Study site and selection of the dogs

Ninety-six dogs (46 males and 50 females, aged 11 months to 15.6 years and weighting 5.4 to 54.8 kg) were enrolled in the present trial. They were privately owned dogs who lived in Dordogne, France. Before inclusion, informed owner consent was obtained. Two veterinary clinics participated to the selection and follow up of the animals. These clinics are located in Vergt and Notre Dame de Sanilhac, two small cities within a zone known to be endemic for *T. callipaeda* [6].

Dogs fulfilling the following inclusion criteria were enrolled in the study: dogs in normal general health, ≥ 7 weeks of age, ≥ 1 kg, not treated with ectoparasiticidal products in the preceding months, and with a history of previous infection by *T. callipaeda* (diagnosed in 2011 by direct examination of nematodes by NS or OP).

#### Study design

In May 2012, included dogs were assigned to one of the three groups, A, B and C, using a random treatment allocation plan. One month before the day of inclusion (D0) a spot on formulation containing 10% imidacloprid and 2.5% moxidectin (Advocate®, Bayer HealthCare Animal Health) was administered to all the dogs in order to be sure that the animals were not infected by *T. callipaeda* at the beginning of the trial. Dogs were allocated to one of three groups (A, B and C). At D0, dogs were checked again for eyeworms and imidacloprid 10% + flumethrin 4.5% collars (Seresto®, Bayer HealthCare Animal Health) were fitted to dogs (n = 36) from group A on the basis of their body weight (i.e., small collar: < 8 kg/large collar: > 8 kg). Dogs (n = 33) from group B received a monthly dose of imidacloprid 10% and moxidectin 2.5% by spot-on dermal application (Advocate®, Bayer HealthCare Animal Health), in accordance with the dogs’ body weight and following the label instructions. Dogs from group B were treated 8 times (from May to December 2012). Dogs (n = 27) from group C were left untreated and served as controls. Dogs from group A and C were examined on days 0, 120 and 240 after inclusion. Dogs from group B were examined every month (just before the application of the spot on) until day 240. During the trial, owners were asked to present their dogs to the consultation as soon as ocular clinical signs occurred. For each dog and each follow-up, the presence of *T. callipaeda* was carefully investigated by ocular examination. Dogs were restrained with a muzzle and the conjunctival fornix inspected for the presence of nematodes using a sterile cotton swab. An approximate count of nematodes number was made for each eye. Nematodes were stored in 70% ethanol and sent to the Parasitology department of Veterinary College of Alfort, France for morphological identification according to Skrjabin *et al*. [17] and Otranto *et al*. [18]. Infected dogs were treated by the application of the moxidectin spot on formulation.

The use of other ectoparasiticides on dogs was not allowed throughout the study period. However, individual treatments (with a product without any repellent activity) were authorized when heavy tick or flea infestations occurred. Due to the nature of the investigational veterinary product (collar), blinding was not applicable.

#### Statistical analysis

The efficacy evaluation was based on the comparison of the incidence of *T. callipaeda*-infected dogs in the two treatment groups (A and B) versus the control group (C). Incidence was calculated as follows: number of dogs newly infected with *T. callipaeda*/(number of negative dogs initially enrolled - number of lost or dead dogs) × 100.

Statistical associations were quantified using prevalence rates of *T. callipaeda* in each of the 3 groups, and were tested using the Chi-2, or Fisher exact (when necessary), statistical test. Factors associated with higher prevalence rates of *T. callipaeda* were also investigated using the Chi-2 or Fisher exact statistical test. Statistical analyses were performed using Epi-Info (Centers for Disease Control and Prevention, USA; World Health Organization, Geneva, Switzerland). Significance level was reached if p-values were < 0.05.

### Results

During the trial, *T. callipaeda* nematodes were detected in 12 (33%) of the 36 collared dogs (group A) whereas no eyeworm could be found in the 33 dogs who received a monthly spot on application of moxidectin (group B). In the control group C, 8 (30%) of the 27 dogs had eyeworms indicating that dogs living in the study area had been naturally exposed to *T. callipaeda* during the summer of 2012. There was no significant difference between the incidence of *T. callipaeda* infection in groups A and C. As a consequence, the slow-release insecticide collar tested in this study did not provide any protection against canine thelaziosis. No signs suggestive of adverse reactions were recorded in treated dogs (either collared or treated with the spot on). Heavy tick or flea infestations never occurred during the trial. As a consequence, no additional insecticide or acaricide treatment was necessary.

**Figure 1:**
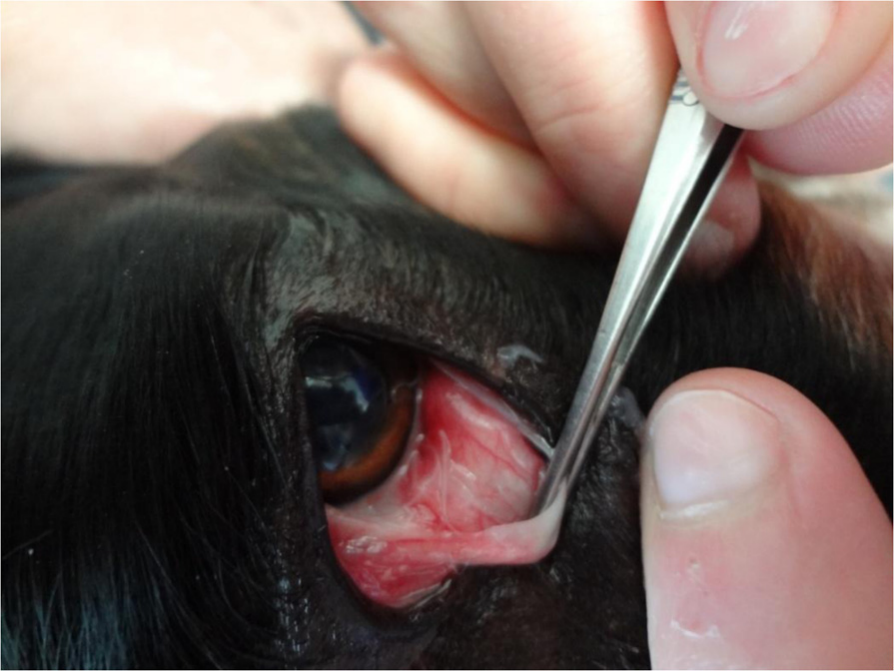
**Conjunctivitis and** ***Thelazia callipaeda*** **specimens in an untreated control dog (Noémie Siméon).**

One month before inclusion (in April 2012), out of the overall 96 dogs, 30 (31%) were already infected by *T. callipaeda*. During the trial, 20 additional cases were detected. Seven dogs were infected twice (before inclusion and during the trial). In 12 cases, no clinical signs were detected. In other cases (n = 38), the following clinical signs were most frequently observed: conjunctival hyperemia (87%), lacrimation (50%), ocular mucopurulent discharge (24%) and chemosis (11%) (Figure 1).

The statistical analysis revealed that the frequencies of the variable parameters were not significantly different among groups (Table 1).

**Table 1:** **Baseline characteristics of groups A (dogs treated with Seresto®), B (dogs treated with Advocate®) and C (untreated dogs)**

**Parameters**	**Group A (n = 36) Seresto®**	**Group B (n = 33) Advocate®**	**Group C (n = 27)**	**P value**
Pure breed, n (%)	26 (72.2%)	26 (78.8%)	16 (59.3%)	0.25
Hair length, n (%)				
Short hair	16 (44.5%)	11 (33.3%)	7 (25.9%)	0.59
Medium hair	12 (33.3%)	14 (42.4%)	14 (51.9%)	
Long hair	8 (22.2%)	8 (24.3%)	6 (22.2%)	
Median age [IQR] (years)	5.7 [3.1-8.5]	5.8 [2.4-9.8]	4.5 [2.8-7.4]	0.97
Male, n (%)	19 (52.8%)	13 (39.4%)	14 (51.9%)	0.48
Contact with other carnivores, n (%)	30 (83.3%)	25 (75.8%)	17 (63%)	0.18
Living in rural area (*versus* city), n (%)	35 (97.2%)	32 (97%)	25 (92.6%)	0.61
Type of living, n (%)				
Farm	9 (25%)	7 (21.2%)	6 (22.2%)	0.98
House	26 (72.2%)	26 (78.8%)	21 (77.8%)	
Flat	1 (2.8%)	0 (0%)	0 (0%)	
Median body weight [IQR] (kg)	24.8 [17.7-29.2]	26.0 [15.8-29.5]	24.5 [19.3-34.1]	0.80

IQR, Interquartile range.

### Discussion

The present study demonstrated that the monthly application of a spot on formulation containing imidacloprid and moxidectin was highly effective in preventing *T. callipaeda* infection in a population of dogs living in an endemic area in France. On the contrary, the slow-release collar tested in this study did not display any protection. To our knowledge, only two trials were performed in order to evaluate the prophylactic activity of antiparasitic drugs against *T. callipaeda*. Rossi *et al*. evaluated the efficacy of a single parenteral administration of moxidectin in naturally exposed dogs in northern Italy [19]. In June 2005, 31 dogs were injected subcutaneously with a sustained-release moxidectin product (at the dose of 0.17 mg/kg) and 32 dogs served as untreated controls. At the end of the trial in January 2006, none of the moxidectin-treated dogs had eyeworms whereas 11 (34%) of the control dogs did. A similar investigation was made to evaluate the prophylactic efficacy of milbemycin oxime [12]. Thirty dogs were treated with oral milbemycin oxime monthly from June to November 2007 with the recommended dose rate for the prevention of heartworm disease and 30 dogs served as untreated controls. One dog in the treated group and 10 dogs in the control group became infected during the trial [12]. As macrocyclic lactones (like moxidectin and milbemycin oxime) have a limited effect on vectors (like the fruit fly *P. variegata*), the prophylactic efficacy of these drugs is accounted by their activity against *T. callipaeda* larvae a short time after they are deposited in the lacrymal secretions by a fruit fly. The therapeutic efficacy of the spot on formulation containing 10% imidacloprid and 2.5% moxidectin was demonstrated in naturally infected dogs in Italy [20]. Elimination of *T. callipaeda* was obtained within 5 or 9 days after treatment.

The use of an insecticide that could limit the exposure of dogs to contaminating fruit flies is another prophylactic strategy. The use of collars impregnated with acaricidal/repellent compounds has provided promising results, indicating that long-lasting protection of dogs against major canine vector-borne diseases can be achieved in most instances [14]. In the present study, the final incidence of thelaziosis in collared dogs and in control animals was not significantly different suggesting that the polymer matrix collar containing a combination of imidacloprid and flumethrin would not provide any protection against canine thelaziosis in field conditions. The amount of active ingredients released by the collar that reach the skin area where the fruit fly uses to land on and the short contact time are probably not sufficient to ensure a repellent activity against fruit flies or to kill them before they are able to feed and transmit *T. callipaeda* larvae.

### Conclusion

As a conclusion, the prevention of canine thelaziosis is possible with a systemic anthelmintic molecule like moxidectin. For dogs living in endemic areas, the monthly administration of systemic macrocyclic lactones throughout the transmission season (summer) should be recommended. For pets spending no more than one month in endemic areas, a single treatment, usually soon after returning home, should be sufficient to assure complete protection.
